# The Assessment of Availability, Formulation, Price and Its Risk Factors of Potassium-Enriched Low-Sodium Salt in China: Implications for Population-Level Salt Reduction

**DOI:** 10.3390/nu18040648

**Published:** 2026-02-16

**Authors:** Dejing Meng, Nicole Ide, Whitney Pyles Adams, Laura K. Cobb, Zeng Ge

**Affiliations:** 1 Department of Health Promotion, China Health Education Center, Beijing 100011, China; mdj-1986@163.com; 2 Resolve to Save Lives, New York, NY 1005, USA; nide@rtsl.org (N.I.); wadams.consultant@resolvetosavelives.org (W.P.A.); lcobb@rtsl.org (L.K.C.); 3 Cardiovascular Health Program, Vital Strategies (USA) Jinan Representative Office, Room 1509, Block B Greentown Financial Center, No. 8 Luoyuan Avenue, Lixia District, Jinan 250000, China

**Keywords:** hypertension, sodium intake, lower-sodium salt substitute, potassium chloride, fiscal policy, China

## Abstract

Background/objectives: Potassium-enriched lower-sodium salt substitutes (LSSS) offer consumers a practical way to increase potassium intake and decrease sodium intake, thereby reducing their risk of high blood pressure and cardiovascular disease. This risk reduction, however, depends on whether consumers can access affordable, evidence-based LSSS products. This study investigated the availability, formulation and price of LSSS in China. Methods: A cross-sectional salt survey was conducted across 195 supermarkets in 15 cities from 2023 to 2025 in China. Results: LSSS availability varied substantially by supermarket size: 90.9% of large (33), 88.9% of middle-sized (45), and only 53.0% of small supermarkets (117) stocked LSSS. Of 1861 total salt products surveyed, 310 were LSSS and 1551 were regular salt. A critical evidence–practice gap exists in product formulation: the mean potassium chloride (KCl) content among unique LSSS products was only 16.6%, with 53.4% of LSSS containing <15% KCl. LSSS products are also consistently more expensive than regular salt. The median LSSS price (11.7 yuan/kg) was significantly higher than regular salt (9.8 yuan/kg, *p* < 0.001). Price disparities were most pronounced at lower price points. Within-brand and within-supermarket comparisons revealed that the lowest-priced LSSS cost 2.0-fold and 2.2-fold more than the lowest-priced regular salt, respectively. Multiple regression analysis identified that LSSS price was significantly associated with KCl content, salt source, supermarket size and geographic region. Conclusions: Consumer access to affordable, effective LSSS products can be increased by expanding LSSS availability in small supermarkets, incentivizing higher-KCl formulations, and reducing price barriers to consumer adoption, which could substantially contribute to salt reduction at the population level.

## 1. Introduction

Globally, approximately 11 million deaths are associated with hypertension annually, with high sodium intake contributing to 1.7 million of these deaths [1]. Salt reduction interventions are considered one of the ‘best buys’ for tackling noncommunicable diseases [2]. Despite the World Health Organization (WHO) setting a global target of 30% relative reduction in population salt intake by 2025 and issuing the SHAKE technical package to guide implementation [3], mean global salt intake remains at 10.8 g/d, more than double the WHO recommendation of 5 g/d [4]. The WHO global report on sodium intake reduction in 2023 shows slow progress with no country reaching the target and proposes potassium-enriched lower-sodium salt substitutes (LSSS) as an innovative dietary measure to reduce salt intake, especially in populations that consume sodium mainly from discretionary salt use added during cooking, such as China [4]. In 2025, the WHO issued guidelines on the use of lower-sodium salt substitutes applicable to discretionary use of salt in the form of table salt to provide guidance for policymakers, program managers, health professionals, and other stakeholders [5].

In China, the prevalence of hypertension is as high as 27.5% [6] and daily salt intake of Chinese adults is approximately 10.5 g (4.12 g sodium) [7]. Salt added during cooking accounts for nearly 70% of salt intake [8]. Dietary potassium intake, which can also help reduce blood pressure, is only 1.5 g/d [7,9], less than half of WHO-recommended levels [10]. Therefore, using LSSS instead of regular salt can both increase potassium intake and reduce sodium intake. Large-scale trials have demonstrated that LSSS with 25% KCl reduces cardiovascular disease (CVD) and all-cause mortality by 13% and 12%, respectively [11]. Scaling up LSSS use could help achieve both the WHO’s salt reduction target and China’s 2030 salt reduction goal and reduce CVD burden globally.

LSSS has been promoted in China with some success. The SMASH project in Shandong province observed a 25% reduction in sodium intake over 5 years, with LSSS sales rising from nearly 0 to 25% of salt sales and systolic and diastolic blood pressure respectively decreasing by 1.8 and 3.1 mm Hg [12]. However, scaling up LSSS use faces key barriers, including low awareness, limited availability, and higher prices. The LSSS market remains poorly understood. A scoping review of the global low-sodium salt market found that LSSS availability was limited, with prices ranging from 1.1 to 14.6 times that of regular salt [13]. One recent survey of online supermarkets in China found that a large number of LSSS products (76) with a wide range of prices were available [14]. A clear understanding of the market for low-sodium salt is critical to the design of policies and interventions to increase the use of low-sodium salt (thereby decreasing salt intake and lowering blood pressure). Therefore, we conducted a supermarket-based market survey to assess the availability, formulation, price of LSSS and the factors associated with LSSS pricing.

## 2. Methods

A supermarket-based, cross-sectional salt survey was conducted in China. Supermarkets were selected as survey points in 15 cities across 10 provinces, including Beijing, Shandong, Heilongjiang, Liaoning, Gansu, Jiangxi, Shanghai, Zhejiang, Jiangsu, and Anhui, from 2023 to 2025. To identify supermarkets for the survey, we first compiled a list of major chain supermarkets in each city through online searches of retail directories and business listings. We prioritized chain supermarkets because they typically stock a comprehensive range of salt brands and products. Baidu Map (a Chinese mapping platform similar to Google Maps) was then used to locate these supermarkets. For chain supermarkets with multiple locations in the same city, only one location was surveyed per chain because they carry identical salt product assortments. To capture the full retail landscape, we also surveyed non-chain retailers, including convenience stores and farmer markets located near the selected chain supermarkets, as these smaller retailers may serve different consumer segments.

At each survey location, photos of the nutrition label and price tag were taken for all salt products. The following information was recorded: supermarket size, chain status (chain or independent), salt source (sea salt, lake salt, or well/rock salt), product name, brand, manufacturer, product code, price, iodization status, potassium and sodium content and package size.

### Variables and Analysis

The primary outcome variable was adjusted price, calculated as the price per kilogram by dividing the retail price by the package size in grams and multiplying by 1000. Salts were classified into price categories based on quartile values of adjusted price (<6.25, 6.25–10, 10–16.57, and ≥16.57 yuan/kg) and labeled price ranges (<1, 1.0–2.0, 2.0–3.0, 3.0–4.0, and ≥4.0 yuan/unit).

Key independent variables included region (north or south), chain status (chain or independent retailer), supermarket size, salt source, iodization status, percent KCl and package size (<200, 200–300, 300–400, and ≥400 g). Salt source refers to the geological origin of the sodium chloride: sea salt (harvested from evaporated seawater), lake salt (from salt lakes), or well and rock salt (from underground deposits). These sources differ in mineral composition and production costs, which may influence pricing. Iodization status indicates whether iodine has been added to the salt to prevent iodine deficiency disorders, a common public health fortification practice in China.

Percent KCl was derived by multiplying the potassium content (mg/100 g) from the nutrition label by 1.91 (the molecular weight of KCl (74.5) divided by the molecular weight of potassium (39)) and then dividing by 1000. We categorized KCl content into the following groups: <15%, 15–20, 20–25, 25–30, and ≥30%.

According to China’s national standard classification of retail format [15], supermarkets are classified as large (≥6000 m^2^), middle (2000–5999 m^2^), and small (200–1999 m^2^), with the small category also including convenience stores and farmer markets with business areas <200 m^2^.

Data are expressed as mean, standard deviation (SD) and median for continuous variables, and as numbers and percentages for categorical variables. Given the right-skewed distribution of salt prices due to some high-priced premium products, we used median prices as the primary comparator between LSSS and regular salt.

We conducted two parallel analyses: one based on all 1861 salt products (including duplicates found across multiple supermarkets) and another based on 585 unique salt products (after removing duplicates). The all-products analysis reflects what consumers encounter when shopping, weighted by how widely each product is distributed. The unique products analysis examines the distinct product offerings available in the market. For unique products, we calculated mean prices weighted by the frequency each product appeared across supermarkets.

We assessed whether median LSSS prices were significantly higher than regular salt prices overall and by product characteristics using Wilcoxon signed-rank tests. We compared the lowest-priced LSSS to the lowest-priced regular salt within each brand (for brands offering both types) and within each supermarket (for supermarkets stocking both types). A multivariate linear regression model with adjusted LSSS price as the dependent variable and independent variables including region, supermarket size, salt source, iodization status, and percent KCl was used to assess relationships of covariates with adjusted LSSS price.

We conducted a sensitivity analysis examining unique salt products with prices weighted by distribution frequency.

All statistical analyses were conducted using SAS software version 9.3. All tests were two-sided, and *p* < 0.05 was considered statistically significant.

## 3. Results

### 3.1. Market Availability of LSSS

A total of 195 supermarkets were surveyed. LSSS availability varied substantially by supermarket size: 90.9% (30/33) of large, 88.9% (40/45) of middle-sized, and 53.0% (62/117) of small supermarkets stocked LSSS. Overall, 1861 salt products from 56 brands were collected, of which 310 (16.7%) were LSSS and 1551 (83.3%) were regular salt. After excluding duplicates, 585 unique salt products remained: 88 (15.0%) LSSS and 497 (85.0%) regular salt.

Half of the brands offered both LSSS and regular salt products. The top five brands accounted for 56% of all unique salt products. The most common packaging material was plastic bags (94.7%). The remaining 5.3% (1 LSSS and 30 regular salt products) came in cans and had high average prices (73 yuan/kg), representing high-end products.

Characteristics of all salt products are presented in Table 1. The largest number of salt products found in a single supermarket was 54 (12 LSSS and 42 regular salt). Although absolute numbers of both LSSS and regular salt were greater in northern provinces and chain supermarkets compared to their counterparts, these differences were not statistically significant. Sea salt and lake salt were the sources for a higher proportion of products than well and rock salt. Among unique products, the proportion of LSSS that was iodized was significantly higher than regular salt (55.8% vs. 43.7%). Package sizes ranged from 82 g to 1000 g. The most common package size for LSSS was 300–400 g (median 350 g), while for regular salt it was ≥400 g (median 400 g).

### 3.2. Potassium Content: The Evidence–Practice Gap

A critical gap exists between evidence-based LSSS formulations and market products. The mean KCl content was 16.1% for all LSSS and 16.6% for unique LSSS, ranging from 10.0% to 29.9%. More than half of LSSS products contained low potassium levels: 57.7% of all LSSS and 53.4% of unique LSSS had <15% KCl. Only 15.5% of all LSSS products contained 25–30% KCl—the range shown to reduce cardiovascular mortality in large-scale trials—and no products exceeded 30% KCl. Products with ≥20% KCl had higher median and mean prices compared to those with lower KCl content (10–20%) (Table 2).

### 3.3. Price Barriers to LSSS Adoption

#### 3.3.1. Overall Price Differences

**Figure 1:**
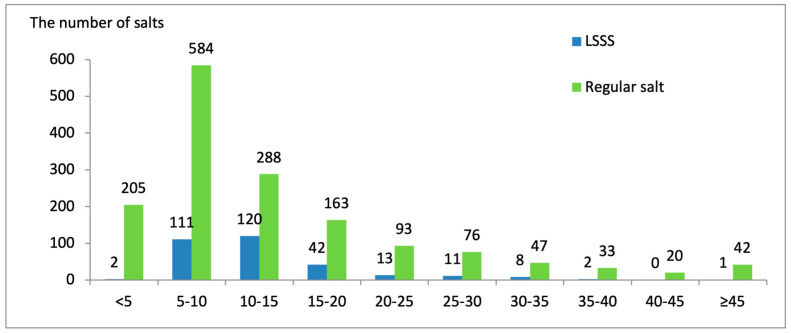
The distribution of the prices (yuan/kg) of LSSS and regular salts.

The labeled price of LSSS ranged from 1.0 to 29.9 yuan per unit, compared to 0.5 to 39.9 yuan for regular salt. Adjusted prices (per kilogram) ranged from 2.86 to 96.14 yuan/kg for LSSS and from 1.25 to 437.8 yuan/kg for regular salt. The distribution of adjusted prices is presented in Figure 1.

Both labeled and adjusted prices of LSSS were consistently higher than regular salt. The median adjusted price was 11.7 yuan/kg for LSSS versus 9.8 yuan/kg for regular salt (Table 3; *p* < 0.001). Price disparities were particularly pronounced at lower price points: only 0.6% (2) of LSSS fell in the lowest price group (<5 yuan/kg) compared to 13.2% (205) of regular salt. Similarly, only 1% of LSSS cost less than 2 yuan per unit, while 10% of regular salt was available at this price point. LSSS products were concentrated in higher price tiers, with 79.6% priced ≥3 yuan per unit compared to 61.6% of regular salt.

#### 3.3.2. Price Differences by Product Characteristics

Price differences between LSSS and regular salt varied by context (Table 3). LSSS prices were significantly higher than regular salt in northern provinces (median: 10.0 vs. 7.5 yuan/kg, *p* < 0.001), but no significant difference was observed in southern provinces. LSSS was more expensive than regular salt in both chain and non-chain supermarkets. By supermarket size, LSSS prices were significantly higher than regular salt in middle- and small-sized supermarkets, but no significant difference was observed in large supermarkets.

Price differences also varied by package size: LSSS was more expensive than regular salt for larger packages (300–400 g and ≥400 g) but not for smaller packages. For iodized products, LSSS was significantly more expensive than regular salt (median: 10.0 vs. 7.1 yuan/kg, *p* < 0.001); however, no price difference was observed between non-iodized LSSS and non-iodized regular salt.

Within the LSSS category, prices varied significantly by characteristics. LSSS was more expensive in southern provinces, chain supermarkets, middle- and large-sized supermarkets, products using sea or lake salt as the source, and iodized products compared to their respective reference groups. Adjusted LSSS prices decreased as package size increased.

#### 3.3.3. Within-Brand and Within-Supermarket Price Comparisons

To assess whether price premiums persist even when consumers can directly compare LSSS and regular salt options, we examined price differences within brands and within supermarkets. Among the 27 brands offering both LSSS and regular salt, the mean price of the lowest-priced LSSS was 9.4 yuan/kg compared to 4.7 yuan/kg for the lowest-priced regular salt—a difference of 4.8 yuan/kg (*p* < 0.05). Among the 132 supermarkets stocking both types, the cheapest LSSS cost a mean of 10.2 yuan/kg compared to 4.7 yuan/kg for the cheapest regular salt—a difference of 5.8 yuan/kg (*p* < 0.05) (Table 4).

### 3.4. Factors Associated with LSSS Pricing

**Figure 2:**
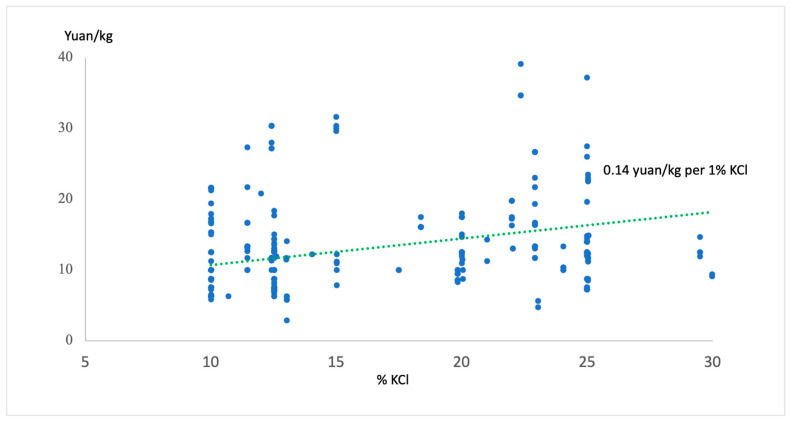
The association of price with % KCl: economic barrier to evidence-based LSSS formulation (SSaSS trial: 25%KCL).

Multiple regression analysis identified several factors significantly associated with LSSS price (Table 5). KCl content was positively associated with price: for every 1% increase in KCl, price increased by 0.14 yuan/kg (*p* = 0.01) (Figure 2). Compared to small supermarkets, LSSS prices were significantly higher in both middle-sized (*p* = 0.003) and large supermarkets (*p* = 0.004). Salt source was strongly associated with price: compared to well and rock salt, sea salt and lake salt are more expensive (*p* < 0.001). However, the price of LSSS is not associated with the iodization status (*p* = 0.16) and regions (*p* = 0.06) after adjustment for multiple factors.

### 3.5. Sensitivity Analysis

Sensitivity analysis results were consistent with the main analysis. For example, the median price of unique LSSS was 3.9 yuan/kg compared to 3.2 yuan/kg for unique regular salt (*p* < 0.001). The median price of iodized LSSS remained significantly higher than iodized regular salt (*p* < 0.001). LSSS price remained significantly associated with region, KCl content, and salt source.

## 4. Discussion

This is the first comprehensive survey of LSSS availability and pricing in Chinese in-person retail markets. We identified 88 unique LSSS products accounting for 15% of all unique salt products across 195 supermarkets in 15 cities. Our findings reveal critical purchasing environment failures that hinder consumer access to this evidence-based cardiovascular intervention and contribute to the development of unhealthy dietary behaviors leading to unbalanced nutrients’ intake: (1) most LSSS products (53.4%) contain <15% potassium levels far below the approximately 25% KCl used in randomized controlled trials (RCTs) shown to reduce mortality, (2) LSSS commands significant price premiums over regular salt, particularly at lower price points where budget-conscious consumers shop, (3) LSSS availability is substantially lower in small supermarkets (53%) compared to large supermarkets (91%), limiting access for populations these stores typically serve, and (4) only half are iodized (dual fortification with both iodine and potassium). To improve consumer uptake of LSSS and reduce dietary sodium intake, a number of key government policies and interventions will be needed.

### 4.1. The Evidence–Practice Gap in Product Formulation

The SSaSS trial found that substitution with LSSS reduced CVD and all-cause mortality by 13% and 12%, respectively, using LSSS with 25% KCl [11]. Most other trials have used similar formulations. A critical disconnect exists between trial evidence and the low percentage of KCl most commonly found in products available in Chinese supermarkets. The mean KCl content of LSSS products was only 16.6%, with 53.4% containing <15% KCl—far below evidence-based formulations. Only 15.5% of products contained 25–30% KCl, and no products exceeded 30% KCl. This also stands in contrast to both Chinese professional standards recommending 20–35% KCl [16,17,18] and the demonstrated feasibility of higher formulations: the Shandong SMASH project successfully promoted 30% KCl products, with 30% KCl LSSS rising from 1% of salt sold to consumers in 2011 to over 25% in 2015 [12].

This evidence–practice gap has direct public health consequences. When most available products contain half or less of the evidence-based potassium dose, the potential cardiovascular benefits will likely be substantially diminished. The gap appears driven by manufacturer decisions rather than consumer acceptance—previous studies in China and internationally have found no taste barriers to 25% KCl formulation [19,20]. Given that China has conducted most LSSS efficacy trials [21] and established policy frameworks supporting LSSS promotion [22,23], aligning market products with evidence-based formulations represents an urgent priority.

### 4.2. Price as a Barrier to Consumer Adoption

Our findings demonstrate that price differences are high enough to potentially limit LSSS adoption, particularly among price-sensitive consumers. The median LSSS price (11.7 yuan/kg) was 19% higher than regular salt (9.8 yuan/kg, *p* < 0.001). Notably, the pricing disparity was most pronounced at lower price points: only 0.6% of LSSS products cost less than 5 yuan/kg compared to 13.2% of regular salt. Direct price comparisons within brands and supermarkets revealed even larger premiums. Among brands offering both products, the cheapest LSSS costs 2.0 times more than the cheapest regular salt (9.4 vs. 4.7 yuan/kg, *p* < 0.05). Within supermarkets stocking both types, the cheapest LSSS costs 2.2 times more than the cheapest regular salt (10.2 vs. 4.7 yuan/kg, *p* < 0.05). These findings contrast with an online marketplace survey that found similar mean prices between LSSS and regular salt (18.2 vs. 16.7 yuan/kg, *p* > 0.05) [14], suggesting that in-person retail channels, where most consumers still purchase salt, face greater price barriers than online channels. This scarcity of budget-friendly LSSS options could effectively exclude price-sensitive consumers from accessing this intervention.

An RCT in rural China demonstrated that eliminating price differences through vouchers substantially increased LSSS adoption (78% vs. 44% in the control group) and sales [24]. This evidence, combined with our finding that higher-KCl products command price premiums (0.14 yuan/kg per 1% KCl increase), indicates that price subsidies could simultaneously address affordability barriers and incentivize manufacturers to increase KCl content.

### 4.3. Pathways to Affordable, Evidence-Based LSSS

Our regression analysis identified potential strategies for producing higher-KCl LSSS at competitive prices. Well and rock salt were the least expensive raw material sources, costing 4.8 yuan/kg less than sea salt and 8.0 yuan/kg less than lake salt. Additionally, adjusted prices decreased with package size. These findings suggest manufacturers could produce evidence-based LSSS (≥25% KCl) using well and rock salt in larger packages without substantial price increases, potentially addressing both the formulation and affordability gaps simultaneously.

Interestingly, iodization status was not associated with LSSS price after adjusting for other factors (*p* = 0.16), despite the proportion of iodized LSSS (173; 55.8%) being significantly higher than regular salt (43.6%). Additionally, the price of LSSS that are dual fortified (both iodized and potassium-enriched) is lower than that of non-iodized LSSS (11.2 vs. 15.3 yuan/kg). These findings indicate that iodine fortification, a WHO-recommended component of salt reduction strategies [3], does not drive price increases and that scaling up LSSS could support rather than hinder iodine deficiency elimination efforts. In addition, higher price of non-iodized salt compared to iodized salt could serve as a positive factor for achieving iodine deficiency elimination.

### 4.4. Equity Implications and Small Supermarket Access

The substantially lower LSSS availability in small supermarkets (53% vs. 91% in large supermarkets) raises equity concerns. Small supermarkets typically serve lower-income neighborhoods and rural areas where cardiovascular disease burden is high. The combination of limited availability, fewer low-cost options, and price premiums creates a double disadvantage for populations who would benefit most from LSSS. Expanding distribution to small retailers and ensuring the availability of affordable, evidence-based formulations in these channels should be priority interventions.

### 4.5. Learning from Successful Implementation

China has demonstrated that large-scale LSSS promotion can succeed when government commitment is paired with market intervention. The Shandong SMASH project achieved remarkable scale-up, increasing LSSS market share from <1% to 25% and reducing population sodium intake by 25% over five years [12]. Importantly, SMASH promoted 30% KCl formulations (double the current market average), demonstrating that evidence-based products can achieve market penetration with appropriate support. The Beijing municipal government’s free sample promotion also successfully increased LSSS availability in chain supermarkets [25].

These successes provide a blueprint for addressing current market failures. Key elements include: government-led promotion campaigns, price interventions to achieve parity with regular salt, requirements or incentives for evidence-based formulations (≥25% KCl), and targeted distribution to underserved channels.

### 4.6. Policy Recommendations

Based on our findings, we recommend a comprehensive market shaping strategy to improve the edible salt environment and increase LSSS use:

1. Implement targeted price subsidies: Prioritize subsidies for evidence-based formulations (≥25% KCl) in small and medium supermarkets. Set a specific target of reducing the LSSS-to-regular-salt price ratio from the current 2.0–2.2-fold premium to ≤1.2-fold at the lowest price points.
2. Expand distribution equity: Mandate LSSS availability in small supermarkets through retail partnership programs or distribution incentives, addressing the current 53% vs. 91% availability gap.
3. Incentivize evidence-based formulations: Provide manufacturing incentives or preferential policies for LSSS using well and rock salt bases with ≥25% KCl and larger package sizes, which our analysis suggests can minimize cost increases.
4. Public awareness campaigns: Couple price interventions with consumer education, highlighting both cardiovascular benefits and the importance of higher-KCl formulations, following the SMASH model and SSaSS trial.
5. Monitor and evaluate: Establish market monitoring systems tracking LSSS market share, KCl content distribution, price trends, and availability by retail channel and geography to guide ongoing policy adjustments.

### 4.7. Strengths and Limitations

This study provides the first comprehensive assessment of LSSS availability and pricing in Chinese in-person retail markets, covering major salt brands across 10 provinces and 15 cities. The large sample (1861 total products, 88 unique LSSS) and systematic data collection across diverse retail channels provide robust evidence for policy development.

Limitations include the lack of sales volume data, which prevents precise market share calculation, though the 15% LSSS proportion among unique products likely approximates actual market share given similar product distribution across stores. Data were collected from urban supermarkets and may not reflect rural market conditions, where previous research suggests LSSS availability and use are lower [26], further highlighting the need for distribution equity interventions, and indicating that the implications at the national level should be formulated with due caution. Given that we could not assess whether price-sensitive consumers actually purchase less LSSS or lower-KCl products, causal conclusions about consumer behavior cannot be drawn from the current data alone, though the RCT evidence on price interventions [24] supports this relationship. Finally, we did not have manufacturer cost data to inform optimal subsidy levels, indicating the need for government-led cost analyses to design effective pricing policies.

## 5. Conclusions

LSSS represents a proven, scalable intervention for reducing cardiovascular disease burden in China, where 70% of sodium intake comes from salt added during home cooking [9]. However, current market conditions create barriers to realizing this potential. Most products contain low potassium levels (mean 16.6% vs. evidence-based approximately 25% KCl), command substantial price premiums (especially at lower price points), and show limited availability in small supermarkets. These market failures are not inevitable—successful government-led interventions have achieved widespread LSSS adoption with evidence-based formulations when market conditions were appropriately shaped [12]. Urgent policy action is needed to implement targeted price subsidies, expand equitable distribution, and incentivize evidence-based formulations. Such interventions would enable consumers to access affordable, effective LSSS products, substantially contribute to achieving China’s 2030 salt reduction goals [23] and improve public health nutrition.

## Figures and Tables

**Table 1:** The characteristics of all salts by LSSS and regular salts.

	LSSS (310)	Regular Salt (1551)	*p*
Region			
North	195 (62.9%)	1003 (64.7%)	0.55
South	115 (37.1%)	548 (35.3%)	
Chain			
Yes	236 (76.1%)	1177 (75.9%)	0.93
No	74 (23.9%)	374 (24.1%)	
Supermarket size			
Large	112 (36.1%)	508 (32.8%)	0.48
Middle	109 (35.2%)	558 (36.0%)	
Small	89 (28.7%)	485 (31.3%)	
Labeled price (yuan/sale unit)			
<1	0 (0.0%)	19 (1.2%)	<0.001
1–2	3 (1.0%)	135 (8.7%)	
2–3	60 (19.4%)	441 (28.4%)	
3–4	117 (37.7%)	349 (22.5%)	
≥4	130 (41.9%)	607 (39.1%)	
Adjusted price (yuan/kg)			<0.001
Q1 (<6.25)	7 (2.3%)	356 (23.0%)	
Q2 (6.25–10)	106 (34.2%)	433 (27.9%)	
Q3 (10–16.57)	134 (43.2%)	358 (23.1%)	
Q4 (≥16.57)	63 (20.3%)	404 (26.0%)	
Ingredient *			
Sea salt	131 (42.3%)	757 (48.8%)	<0.001
Well and rock salt	139 (44.8%)	553 (35.7%)	
Lake salt	18 (5.8%)	203 (13.1%)	
Package size (g)	350	400	<0.001
Package size (g)			
<200	0 (0.0%)	37 (2.4%)	<0.001
200–300	44 (14.2%)	204 (13.2%)	
300–400	126 (40.6%)	393 (25.3%)	
≥400	140 (45.2%)	917 (59.1%)	
Iodized			
Yes	173 (55.8%)	677 (43.6%)	<0.001
No	137 (44.2%)	874 (56.4%)	

* 60 missing values because of undefined salt ingredient. Data are expressed in the number of products (%).

**Table 2:** The price with the percent of KCl.

	All LSSS (310)			*p*	Unique LSSS (88)			*p*
	N (%)	Mean (SD)	Median		N (%)	Mean (SD)	Median	
KCl		16.1 (6.1)	12.5			16.6 (6.1)	13.0	
KCl (%)								
<15	179 (57.7)	11.4 (6.1)	10	<0.001	47 (53.4)	11.7 (5.5)	11.4	0.03
15–20	22 (7.1)	11.8 (3.3)	10		7 (8.0)	11.3 (2.4)	10.9	
20–25	61 (19.7)	16.6 (7.2)	14.3		21 (23.9)	15.7 (7.7)	13.3	
25–30	48 (15.5)	14.6 (12.8)	12.4		13 (14.8)	19.5 (23.3)	12.5	
≥30	0				0			

**Table 3:** The prices of salts by LSSS and regular salts.

	LSSS		RS		*p*
	Mean (SD)	Median	Mean (SD)	Median	
Number of products	310		1551		
Adjusted price (yuan/kg)	13.0 (7.9)	11.7	17.5 (41.0)	9.8	<0.001
Region *					
North	11.6 (8.1)	10.0	14.5 (33.3)	7.5	<0.001
South	15.4 (6.7)	13.3	22.9 (51.7)	13.0	0.08
Chain *					
Yes	14.3 (8.5)	12.5	20.6 (46.5)	11.3	0.028
No	8.9 (2.8)	8.8	7.7 (4.8)	7.5	<0.001
Supermarket size *					
Large	14.3 (10.3)	12.0	21.6 (43.2)	12.3	0.68
Middle	14.1 (6.4)	12.7	18.1 (40.8)	10.0	0.002
Small	9.9 (4.1)	8.8	12.6 (38.3)	7.5	<0.001
Ingredient *					
Sea salt	15.6 (7.0)	13.3	24.7 (56.9)	11.7	0.011
Lake salt	19.0 (6.1)	16.1	15.5 (10.9)	12.5	0.003
Well and rock salt	9.3 (3.0)	8.8	9.0 (8.8)	7.3	<0.001
Package size (g) *					
<200			201.3 (177.0)	145.6	
200–300	20.8 (7.7)	17.1	21.9 (14.4)	19.4	0.77
300–400	14.3 (8.9)	12.7	13.2 (6.2)	11.7	0.04
>400	9.3 (3.7)	7.8	10.9 (11.2)	7.3	<0.001
Iodize ^*^					
Yes	11.2 (6.2)	10.0	9.6 (9.6)	7.1	<0.001
No	15.3 (9.1)	13.3	23.6 (53.1)	12.5	0.23

* *p* < 0.001 for comparisons of the price of LSSS by different characteristics.

**Table 4:** Price differences between LSSS and regular salt.

	n	LSSS	RS	Difference
Salt brands *	27	9.4 (4.2)	4.7 (3.3)	4.8 (3.9)
Supermarkets	132	10.2 (4.9)	4.7 (3.2)	5.8 (4.7)

* One high-end brand excluded.

**Table 5:** Results of multiple stepwise regression analysis for LSSS.

Independent Variable	Coefficients	Std. Error	t	*p*
Region				
North	Reference group			
South ^a^	1.36	0.71	1.9	0.06
Iodized				
Yes	Reference group			
No	0.95	0.68	1.4	0.16
KCl	0.14	0.05	2.53	0.01
Supermarket size ^b^				
Small-sized supermarket	Reference group			
Middle-sized supermarket	2.38	0.80	2.99	0.003
Large-sized supermarket	2.32	0.80	2.91	0.004
Ingredients ^c^				
Well and rock salt	Reference group			
Sea salt	4.76	0.73	6.49	<0.001
Lake salt	7.98	1.34	5.97	<0.001

^a^ including Jiangxi, Shanghai, Zhejiang, Jiangsu, and Anhui; ^b^ supermarkets classified with different business areas: large-sized (≥6000 m^2^), middle-sized (2000–5999 m^2^), and small (≤1999 m^2^); ^c^ sea salt (harvested from evaporated seawater), lake salt (from salt lakes), or well and rock salt (from underground deposits).

## Data Availability

The data that support the findings of this study are available from the corresponding author upon reasonable request.
